# Radar micro-Doppler signatures of drones and birds at K-band and W-band

**DOI:** 10.1038/s41598-018-35880-9

**Published:** 2018-11-26

**Authors:** Samiur Rahman, Duncan A. Robertson

**Affiliations:** University of St. Andrews, SUPA School of Physics & Astronomy, North Haugh, St. Andrews, KY16 9SS Fife Scotland

## Abstract

Due to the substantial increase in the number of affordable drones in the consumer market and their regrettable misuse, there is a need for efficient technology to detect drones in airspace. This paper presents the characteristic radar micro-Doppler properties of drones and birds. Drones and birds both induce micro-Doppler signatures due to their propeller blade rotation and wingbeats, respectively. These distinctive signatures can then be used to differentiate a drone from a bird, along with studying them separately. Here, experimental measurements of micro-Doppler signatures of different types of drones and birds are presented and discussed. The data have been collected using two radars operating at different frequencies; K-band (24 GHz) and W-band (94 GHz). Three different models of drones and four species of birds of varying sizes have been used for data collection. The results clearly demonstrate that a phase coherent radar system can retrieve highly reliable and distinctive micro-Doppler signatures of these flying targets, both at K-band and W-band. Comparison of the signatures obtained at the two frequencies indicates that the micro-Doppler return from the W-band radar has higher SNR. However, micro-Doppler features in the K-band radar returns also reveal the micro-motion characteristics of drones and birds very effectively.

## Introduction

Commercial drones have gained huge popularity in recent times due to their ease of operation, robust performance and low cost. Considering the various applications a drone can be used for, it has been predicted by the Federal Aviation Association (FAA) in 2016 that the drone usage will be tripled by 2020^1^. Along with their advantages, the easy availability of drones also poses significant security threats. Small commercial drones (categorised by the CAA as ‘small unmanned aerial vehicles’ weighing <20 kg, which typically range from 30 cm to 1 m in width) can be used for contraband or explosive material transportation, illegal video streaming and taking images of critical national infrastructure. Even the mere presence of a drone near an aircraft can have catastrophic consequences. As this is quite a recent phenomenon, the regulation of drone flying is an ongoing challenge. In order to properly regulate, it is at first necessary to have sufficient technology for drone detection and tracking in the airspace. Straightforward adoption of existing airspace surveillance systems will not suffice as drones are much smaller in physical size and will be flying slowly at lower altitudes. NASA is currently exploring the technologies available which can eventually be incorporated into a well-defined Unmanned Aircraft System (UAS) traffic management protocol^2^.

A conventional air surveillance radar system (operating usually at L-band or S-band) can rely on the radar cross section (RCS) of an aircraft for detection, but this may not always provide reliable detection in case of drones. Even if a dedicated system is built to be sensitive enough to detect a small object like a drone, just RCS information is not adequate. Birds have similar physical size to drones and also will fly at similar altitudes and speeds. A reliable drone detection radar system must have the capability to discriminate between a bird and a drone. The study of micro-Doppler signatures is hence of great importance in this context. Micro-Doppler is generated due to the micro motion of the various components within a target^3^. In the case of a drone, it is generated by the propeller blade rotation and for birds it is due to the wings flapping. Along with the bulk Doppler due to the target’s radial velocity, micro-Doppler signatures can be measured with radar which is coherent (i.e. the signal phase change is in direct correlation with the target motion). A number of research works have been conducted to address this topic in recent years. In^3–5^, the mathematical groundwork of the micro-Doppler phenomenon in the context of radar has been laid out. The mathematical models can be used to predict how the micro-Doppler properties of the target modulate the radar return signal. Further research has been carried out using this foundational work to study radar micro-Doppler signatures of drones and birds, but mainly in X-band or lower frequencies^6–10^. Using a higher frequency has the advantage of better Doppler resolution in a shorter time which would allow the fine features to become more evident^11^. Alternatively, for a given integration time, a higher frequency radar will achieve a higher Doppler resolution than a lower frequency radar, thus revealing finer Doppler features. Whilst micro-Doppler measurements of the wingbeats of insects using millimetre wave radar have previously been reported^12^, we are not aware of any report so far on the experimental results of bird micro-Doppler at both K-band and W-band, simultaneously. There are few reports published on K-band micro-Doppler data. In^13^, micro-Doppler measurement results of Peregrine falcon and Serotine bat have been reported using 24 GHz coherent Continuous Wave (CW) radar. That radar was quite short range as the maximum detection range for a Peregrine falcon was only about 50 metres. Those results showed the frequency modulation in the Peregrine spectrogram due to wing beat but the wing beat modulation was not observed for the bat. In^14^, radar signature analysis of a Mallard duck at K-band was reported, showing a spectrogram of the flying duck, revealing the wing beats, obtained using a 24 GHz CW radar. In^15^, micro-Doppler signatures of various types of drones were reported at Ka-band (35 GHz). That work compared the drone micro-Doppler signatures with simulated bird micro-Doppler signatures, as no experimental data for the birds were taken. In^16^, a 24 GHz radar system performance evaluation is reported by illustrating drone micro-Doppler results. The experimental scenario was static in the sense that the drone was hovering at a very short distance away from the radar, but the paper successfully proves the concept of using the radar system for drone micro-Doppler feature extraction. Similarly, a custom built 24 GHz coherent FMCW radar was reported^17^, which shows preliminary results of the spectrogram plots of drone and bird micro-Doppler. At W-band, along with reporting the initial results of drone micro-Doppler signatures^11^, we have shown analysis of the signal processing aspects of the drone micro-Doppler signature extraction using combination of Short Time Fourier Transform (STFT), Continuous Wavelet Transform (CWT) and Discrete Wavelet Transform (DWT) methods^18^. Our review of the literature suggested that there has been no reports on the following topics: a) bird micro-Doppler signature at K-band where instead of a single bird, birds of different sizes have been used for better evaluation of the micro-Doppler characteristics, b) bird micro-Doppler signature analysis at W-band, c) report of micro-Doppler signatures of different sizes of drones at W-band, d) simultaneous experimental data collection at K-band and W-band for direct comparison of the micro-Doppler features at these two different frequency bands. The objective of this work is to create an extensive dataset of drones and birds at these high frequency bands, covering all the topics mentioned above. It should also be noted that most of the current drone detection radar systems operate in lower frequency bands, which makes the radar sensor bulky. There is a need for portable, compact and low-cost radar systems for this purpose. As the automotive industry uses these two bands (24 GHz and 77 GHz) for car radar, there are low cost RF components available in the market at these frequencies, enabling one to design and build a low-cost radar system.

Along with presenting bird micro-Doppler results at K-band and W-band simultaneously for the first time, this paper also reports on the experimental results of drone micro-Doppler at the same two frequency bands. For a low power system like that we have used during data collection (transmit power about 200 mW and antenna gain of about 33 dBi), the K-band radar will have a range coverage for a typical drone or bird (RCS approximately −20 dBsm) with a 10 dB SNR of about 700 m, for a single shot measurement. In contrast, a W-band radar with similar parameters will have a range coverage of about 380 m. Even though higher frequency radars may have reduced range coverage, they offer other attributes which are advantageous from an end-user’s perspective. Hence, W-band might be chosen as it will require a small antenna, thus yielding a very compact system. On the other hand, a K-band system may be a very good trade-off between longer ranges at the expense of a physically larger system whilst leveraging low cost radar chipsets. To investigate thoroughly the micro-Doppler characteristics at these frequency bands, different types of drones and birds have been used. The results shown in this paper clearly demonstrate the characteristic micro-Doppler properties of drones and birds separately, at K-band and W-band, as well as how the properties compare and contrast with respect to each other. It should be noted that whilst the data also contain information on the absolute radar cross section (RCS) of the targets, as all the radars were very well calibrated, the detailed analysis of their RCS is reported in a separate publication^19^. This paper hence exclusively focuses on the micro-Doppler results from the experimental trials.

## Methods

### Concept of radar micro-Doppler signature extraction

Micro-Doppler is produced by the periodic movement of any structural component of an object^20^. The periodic movement creates micro-motion, which in turn induces side-bands about the bulk Doppler frequency. The phase of the radar return signal from such an object (e.g. human walking, bird or drone flying) will change accordingly. Hence, if the radar is coherent, the change in phase values of consecutive pulses, in pulsed radar, or consecutive chirps, in Frequency Modulated Continuous Wave (FMCW) radar, will directly correspond to the change in Doppler. Either a range-Doppler plot or a velocity-time spectrogram can be produced from the data to visualise and analyse the micro-Doppler features. In FMCW radar, a 2D Fast Fourier Transform (FFT) is performed on the data to obtain Doppler information^21^. At first an FFT is performed on every chirp which gives the range profile. Then for a given range bin, a second FFT is performed on a number of consecutive chirps. Generally, the Short Time Fourier Transform (STFT) is used to obtain these plots^22^, as unlike the Fourier Transform, the STFT provides temporal information along with frequency information. This is done by segmenting the dataset (by windowing) and performing Fourier transform of the segments sequentially. Varying the window length changes the frequency and temporal resolution simultaneously^5^ (one decreases when the other increases). The amount of Doppler information contained in the radar data depends on the hardware sampling capability. In the case of FMCW radar (as have been used here), the maximum unambiguous Doppler frequency is *f*_*d, max*_ = *1/2t*_*s*_, where *t*_*s*_ is the chirp period. The Doppler frequency generated by drone propeller will be much higher than the frequency generated by bird wingbeat. A commercial drone can have a propeller rotation rate of order 100 Hz typically (it changes to maintain orientation and vary speed). The maximum Doppler shift can be calculated as *((4πLΩ)/λ) cosβ*, where *L* is the length of the blade from its centre, Ω is the rotation rate in revolutions/second, *β* is the elevation angle with respect to the radar and *λ* is the radar wavelength. Considering the physical configuration of a commonly used drone (DJI Phantom 3 Standard, blade length is 13 cm) and for zero-degree elevation angle, the maximum Doppler frequency for a 100 Hz rotation rate would be 50 kHz for a 94 GHz operating frequency and 13 kHz for 24 GHz.Satisfying Nyquist’s criterion, the required chirp period to unambiguously sample the Doppler in this case will be approximately 10 μs at 94 GHz and 38 μs at 24 GHz. Such a short chirp period is very demanding for the radar hardware and processor, so a longer chirp period was used in practice. The chirp period was chosen in both frequency bands to fully sample the maximum expected bulk velocity Doppler from both drones and birds during data collection, which is approximately 10 ms^−1^, with the acceptance that the propeller micro-Doppler would be aliased. At 94 GHz, the chirp period was 80.5 μs (T-220) and at 24 GHz it was 234.8 μs. These values correspond to chirp repetition frequencies (CRF) of 12.4 kHz and 4.25 kHz, respectively. The CRF value can be converted to obtain velocity, −(CRF * *λ)/2)*. This gives a maximum unambiguous velocity range of ±9.93 ms^−1^ at 94 GHz and ±13.3 ms^−1^ at 24 GHz. Whilst this will not fully sample the drone micro-Doppler it will still yield a very reliable signal for analysis and classification. Unambiguously sampling the rotor blade signals will provide repeatable characterisation of a specific drone. It will have information regarding the rotor rotation rate which theoretically can be used to characterise the type of drone (i.e. if it is a quadcopter or a hexacopter). In practice, it is actually not that simple as the individual rotor blades rotate at different speeds, hence the resultant modulation becomes more complicated^11^. When the micro-Doppler of the rotor blade is under-sampled, it does not reveal the blade flashes; instead a spread about both sides of the bulk Doppler on the Doppler axis is seen, which is going to be demonstrated in the results section. This can be used as a very useful feature for drone classification as it is a unique characteristic of drone micro-Doppler, not present in a bird micro-Doppler plot. The bird wing flapping frequency is much lower (maximum value is around 10 Hz for the birds used here) so radars operating with these CRFs will be able to fully resolve the bird micro-Doppler.

### Experimental scenario and equipment

**Figure 1:**
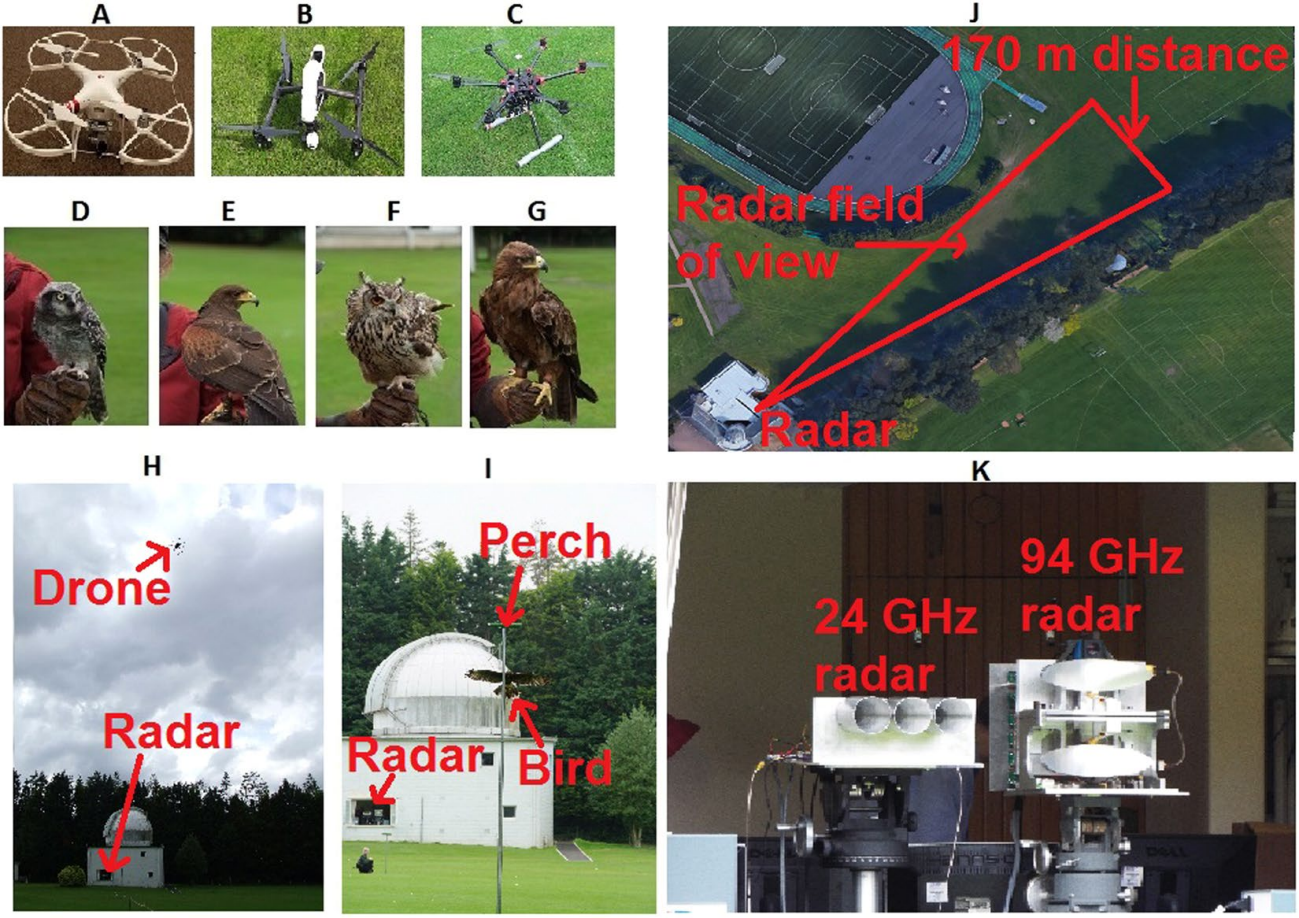
Targets used during data collection and the experimental scenario. (**A**) DJI Phantom 3 Standard; (**B**) DJI Inspire 1; (**C**) DJI S900 Hexacopter; (**D**) Northern Hawk Owl; (**E**) Harris Hawk; (**F**) Indian Eagle Owl; (**G**) Tawny Eagle. (**H**) Drone data collection scenario; (**I**) Bird data collection scenario; (**J**) Deployment area at the University of St Andrews observatory (**K**) K-band and W-band coherent radar systems used for data collection.

The data collection was done in two parts. One part consisted of flying three different models of drones while taking data with both K-band and W-band radar systems. In the second part, four different birds of prey flew between perches under the command of the falconers and again both radars collected data. All the bird handling during the experiment had been done by the professional falconers of ‘Elite Falconry’. The handling and the experimental protocols were carried out in accordance with the relevant guidelines and regulations approved by the Scottish Society for Prevention of Cruelty to Animals (Scottish SPCA), the Scottish Hawk Board and the Fife Council performing animals license. All targets flew back and forth in a radial direction so that the radars could capture the radial velocity in both directions. As can be seen in Fig. 1(H,I), the two radars were looking out from the laboratory window while the targets flew over an open grassy area. As can be seen in Fig. 1(J), the maximum range during the experimental trial is about 170 m beyond which the measurements are affected by clutter from trees and the ground. The available elevation angle is limited to about 25° caused by obscuration from a window frame. Two quadcopters and a hexacopter were used, with the following details:

- *DJI Phantom Standard 3* (weight 1.216 kg, width 35 cm, blade length 13 cm)
- *DJI Inspire 1* (weight 2.845 kg, width 58 cm, blade length 34.5 cm)
- *DJI S900* (weight 3.3 kg, width 90 cm, blade length 38.1 cm)

Four birds of different sizes were used with the following details:

- *Northern Hawk Owl* (weight 0.26 kg, length 40 cm, wingspan 45 cm)
- *Harris Hawk* (weight 0.71 kg, length 55 cm, wingspan 115 cm)
- *Indian Eagle Owl* (weight 0.97 kg, length 52 cm, wingspan 135 cm)
- *Tawny Eagle* (weight 1.84 kg, length 65 cm, wingspan 175 cm)

It should be noted that the radar system must be phase coherent to measure Doppler. For W-band measurements, a 94 GHz, very low phase noise, coherent FMCW radar, called T-220 has been used^23^. It has a homodyne architecture with dual fan beam antenna (0.9° azimuth and 3° elevation beamwidths, 40.5 dBi gain, circular polarization). The transmit power is +18 dBm. Another FMCW radar with a heterodyne architecture called NIRAD^24^ was used on one occasion to obtain drone data using a short chirp period (20 μs). This is also a low phase noise, coherent, 94 GHz radar. NIRAD has a single pencil beam antenna (0.74° azimuth and 0.8° elevation beamwidths, 42.5 dBi gain, circular polarization) with +20 dBm transmit power. For the phase coherent K-band FMCW radar, a 24 GHz radar evaluation board from Analog Devices was used. Three 24.5 dBi gain horn antennas (11.2° azimuth and 11.2° elevation beamwidths) were designed and built in house. A power amplifier was added to the transmit port of the evaluation board to boost the transmit power to +25 dBm. It should be noted that the maximum power density level during the experiment is very low and is not harmful (0.0338 mW/cm^2^ at 1 m). During data collection, the chirp bandwidth of both radars was set to 150 MHz, giving a 1 m range resolution. Frequency modulation in the 24 GHz system uses a phase locked loop (PLL) controlled voltage controlled oscillator (VCO), whilst the 94 GHz radar uses an upconverted and multiplied direct digital synthesis (DDS) chirp source. The control codes of both the radars are written in National Instruments LabWindows/CVI which enables live control, data display and data saving during data collection.

## Results

### Drone micro-Doppler

**Figure 2:**
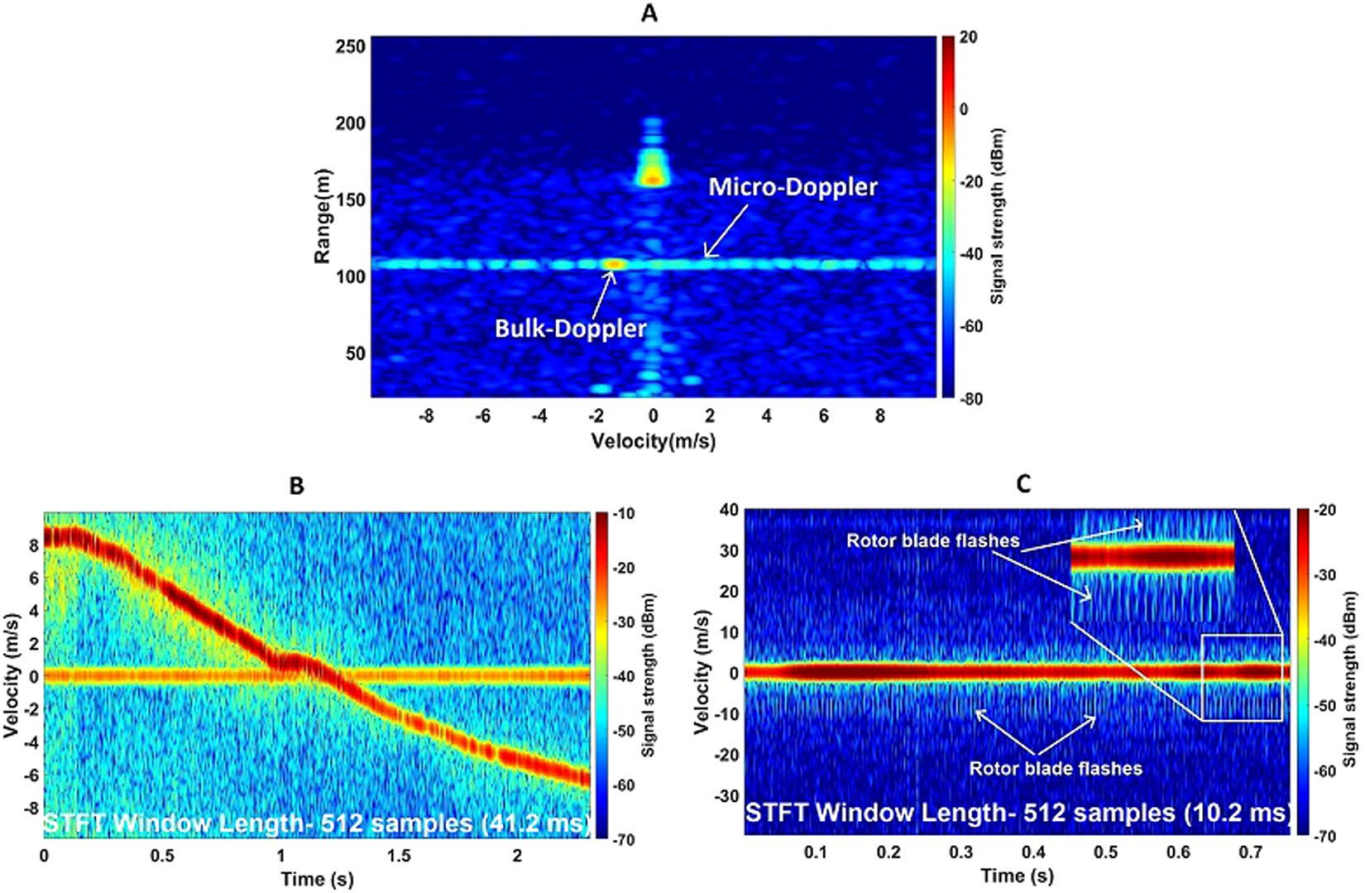
Drone range-Doppler plot and spectrogram plot showing micro-Doppler and rotor blade flashes. (**A**) Example of a range-Doppler profile of a drone flying at ~110 m range showing Doppler features. The large zero-Doppler target at ~170 m is a trihedral; (**B**) Spectrogram of a flying (99–114 m range) DJI Phantom 3 Standard at 94 GHz where micro-Doppler signals are seen about the bulk-Doppler; (**C**) Spectrogram of DJI Phantom 3 Standard at ~45 m range at 94 GHz with shorter chirp period (20 μs) revealing blade flashes.

For the live update during data collection, range-Doppler profiles are produced by processing 128 consecutive chirps, corresponding to window lengths of 10.3 and 30 milliseconds for 94 GHz and 24 GHz respectively. This is very useful to ensure that the target is well within the radar beam during its flight. Figure 2(A) shows how a drone appears on a range-Doppler profile. The drone exhibits clearly visible micro-Doppler, with strength of about −40 dBm which is 40 dB higher than the noise floor. Figure 2(B) is a spectrogram plot of a DJI Phantom 3 Standard at 94 GHz, obtained by using an STFT window length of 41.2 milliseconds. The negative velocity corresponds to the target coming towards the radar and vice versa so the DJI Phantom in the spectrogram was first going away from the radar and then changed direction at around 1.25 s. The spectrogram is obtained by only selecting the range bins occupied by the drone. The drone was about 120 m away from the radar.

The micro-Doppler spread is again clearly visible and is around 30 dB lower than the bulk Doppler. This is expected as the micro-Doppler signals are produced by the smaller plastic rotor blades, whereas the bulk Doppler arises from the larger main body. When the rotor blade faces the radar, they produce a flash which can be seen on the spectrogram obtained with higher Doppler sampling rate (to capture the fast rotation). The spectrogram seen in Fig. 2(C) is produced by changing the chirp period of the 94 GHz NIRAD radar to be 20 μs. This increases the velocity coverage to ±40 ms^−1^. The vertical streaks in the spectrogram correspond to rotor blade flashes. As all the four rotors have different speeds to maintain orientation, the spacing between the consecutive flashes is not constant. The DJI Phantom was flying at 10 ms^−1^ speed in this case and very quickly disappeared from the range bin selected here hence the signal strength degrades after about 0.2 seconds.

**Figure 3:**
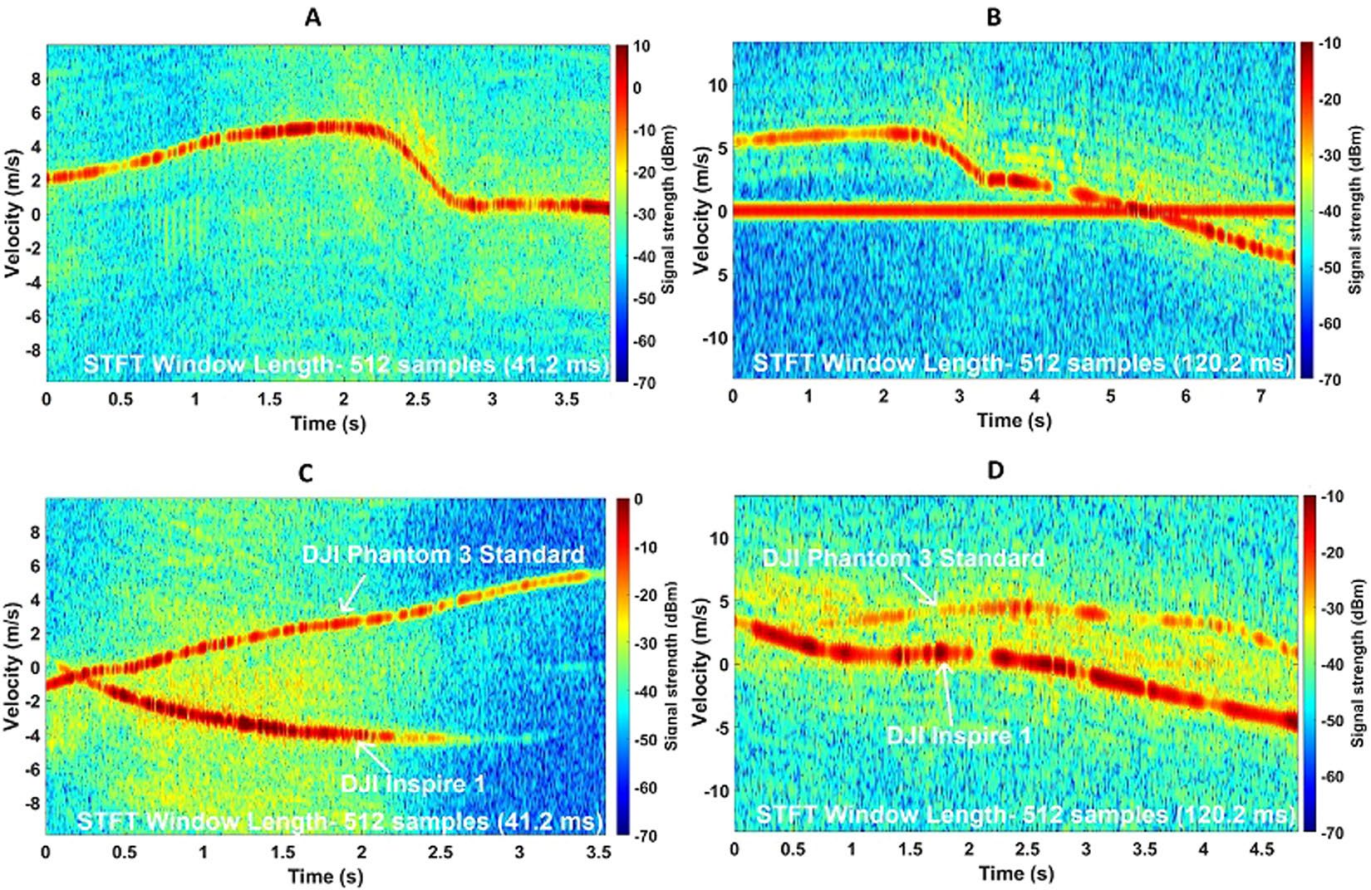
DJI Phantom 3 Standard and DJI Inspire 1 spectrogram plots showing micro-Doppler features at both K-band and W-band. (**A**) Spectrogram of a flying DJI Inspire 1 at ~85 m range at 94 GHz; (**B**) Spectrogram of a flying DJI Inspire 1 at ~85 m at 24 GHz; (**C**) Spectrogram of DJI Phantom 3 Standard (~75 m range) and DJI Inspire 1 (~85 m range) flying together at 94 GHz; (**D**) Spectrogram of DJI Phantom 3 Standard (~75 m range) and DJI Inspire 1 (~85 m range) flying together at 24 GHz.

Spectrogram plots of the DJI Inspire 1 can be seen in Fig. 3. The drone was flying at about 85 m away from the radar. Figure 3(A) is obtained by processing the 94 GHz radar data with an STFT window length of 41.2 milliseconds (512 samples). The same number of samples was used when processing the 24 GHz radar data which in this case correspond to a window length of 120.2 milliseconds. A longer window length increases the frequency resolution which reveals helicopter rotor modulation (HERM) lines^11^ in the spectrogram plot. These are the approximately horizontal lines roughly parallel to the bulk-Doppler trace in Fig. 3(B). The Phantom and the Inspire were flown simultaneously to analyse the micro-Doppler features in the presence of multiple targets. In Fig. 3(C), the drones are flying in opposite directions: the Inspire (bottom) is flying towards the radar whereas the Phantom (top) is moving away. The bulk-Doppler signal strength is about 10–15 dB stronger for the Inspire which is expected as it is bigger in size, although it should be noted that the signal strength will vary quite a lot depending on range, orientation and how well within the radar beam the drones are. The micro-Doppler return is also stronger for the Inspire in this case. In Fig. 3(D), the processed spectrogram is of 24 GHz data. Here, the drones were about 85 m away from the radar and were flying at slightly different altitudes. Again, the signal return from the Inspire (bottom) is stronger and significant micro-Doppler contributions are also observed.

**Figure 4:**
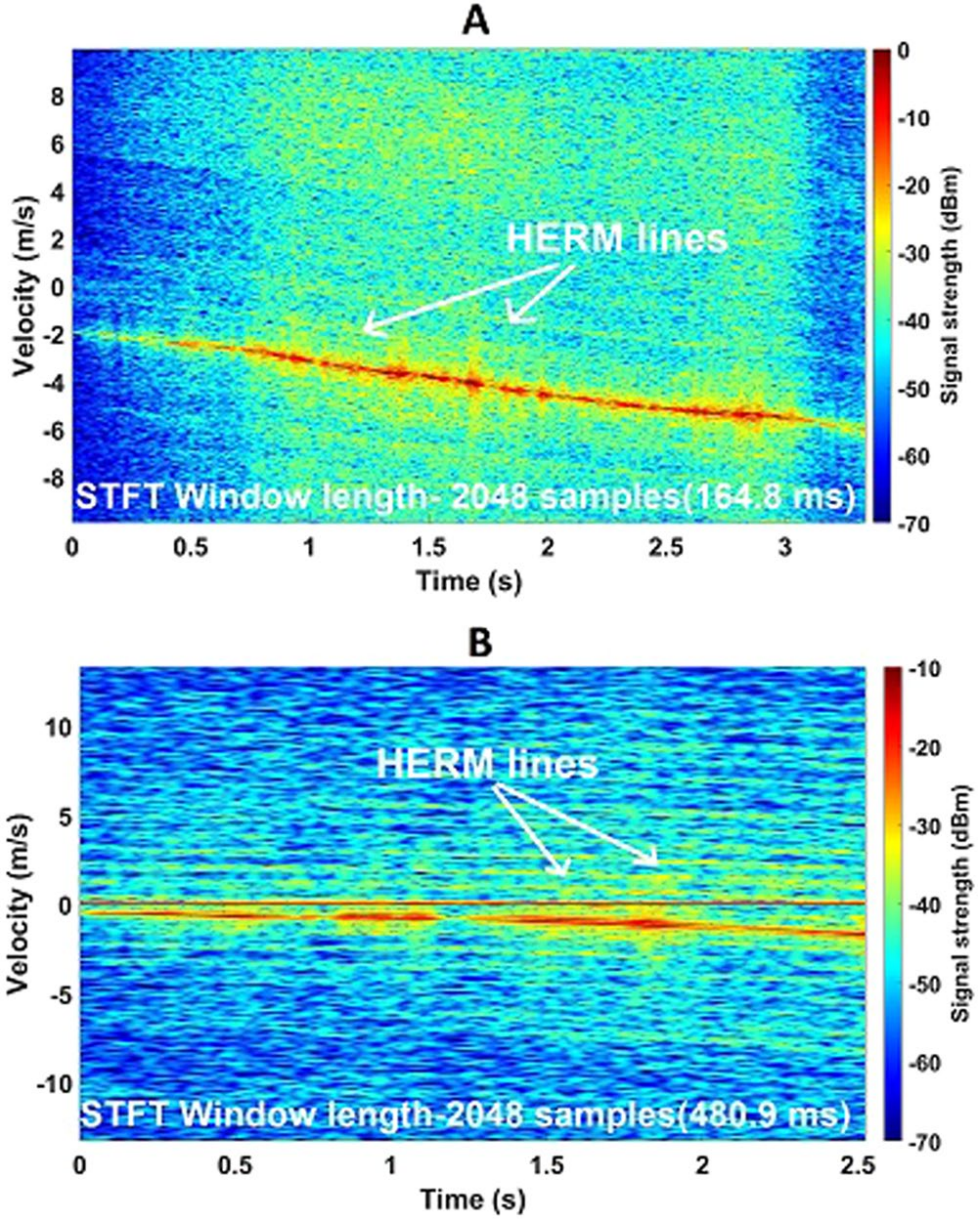
DJI S900 Hexacopter spectrogram plots showing micro-Doppler features at both K-band and W-band. (**A**) Spectrogram of a flying DJI S900 Hexacopter at ~95 m range at 94 GHz; (**B**) Spectrogram of a flying DJI S900 Hexacopter at ~95 m range at 24 GHz.

Figure 4(A,B) illustrate that the micro-Doppler return from the DJI S900 Hexacopter is the strongest of all the drones tested which is understandable as the blades of the hexacopter are biggest and it has the most number of rotor blades. For the STFT spectrogram, 2048 samples have been used which correspond to 164.8 milliseconds for 94 GHz and 480.9 milliseconds for 24 GHz. The micro-Doppler spread in both cases occupies the entire velocity-Doppler axis. HERM lines are more apparent in the 24 GHz plot which is mainly because of the longer window length during processing. The HERM line spacings are not constant as the contributions from six propellers rotating at different speeds do not add up add up in a deterministic way.

All the radar measurements are receiver noise limited. The noise floors appear in the FFTs with mean values of −77.5 dBm for the 24 GHz radar, −73.4 dBm for T-220 and −62.2 dBm for NIRAD, although the latter varies across the range profile. All the micro-Doppler plots with minimum colour scale values which exceed these noise floors. As seen from the drone micro-Doppler plots, the absolute return power from the main body falls within the range of 0 dBm to −20 dBm for ranges of approximately 70–120 m. The return signal strength from the propeller blades is approximately 20–40 dB lower than the main body return. It should be noted that the variation is heavily dependent on the aspect angle of the blades with respect to the radar. This confirms that the radar systems were sensitive enough to capture the blade contributions at these ranges with at least 10 dB SNR.

### Bird micro-Doppler

Collecting bird data for micro-Doppler analysis was more difficult than drone data collection because the flight path of birds cannot be entirely controlled. A trained bird can be made to fly from one perch to another for bait, but it may take a slightly different route every time, depending on wind conditions or its mood. Also, the birds used did not always flap their wings. Typically, they flapped their wings during take-off and landing and glide in the middle to conserve energy. Also, as they fly quite close to the ground, the radar receives ground clutter along with the signal from the target, especially in the case of 24 GHz radar which has a wider elevation beamwidth. Nonetheless, it was a very successful experimental providing a lot of micro-Doppler data. Again, live updates on the range-Doppler profiles were used to ensure that the saved data contained micro-Doppler information.

**Figure 5:**
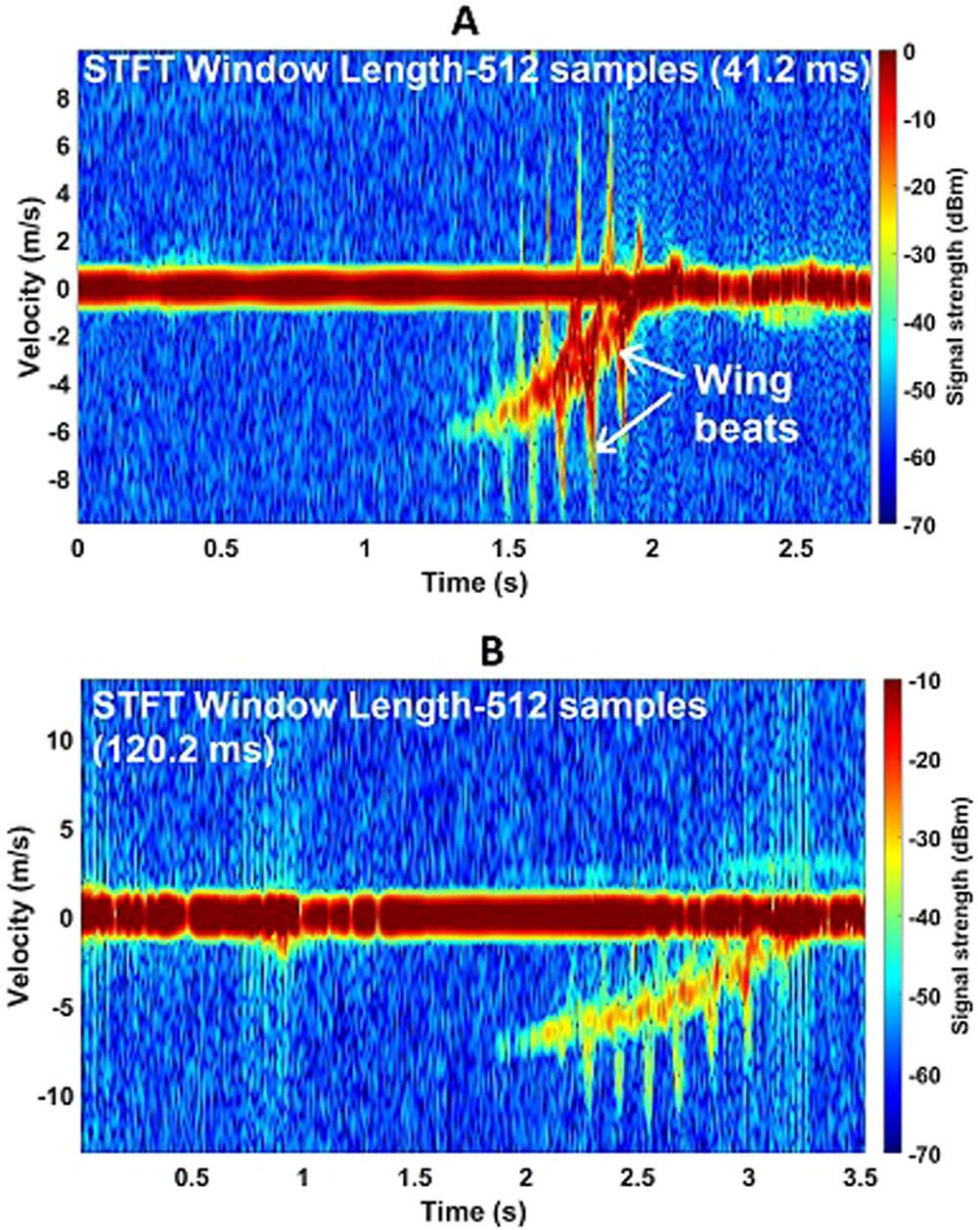
Northern Hawk Owl spectrogram plots showing micro-Doppler features at both K-band and W-band. (**A**) Spectrogram of Northern Hawk Owl approaching the perch at 30 m away from the radar at 94 GHz, exhibiting wing beats; (**B**) Spectrogram of Northern Hawk Owl approaching the perch at 30 m away from the radar at 24 GHz.

Figure 5(A,B) shows the spectrograms of a Northern Hawk Owl. During its flight, it took a sideways trajectory to approach the perch, hence was initially out of the radar beam. The radar was pointed at the destination perch so the bird appeared in the beam when it was landing. During deceleration it started to flap its wings, which is captured by both radars. The wingbeat frequency is approximately 6 Hz here. The wingbeat velocity ranges from 5–10 ms^−1^ (by measuring the micro-Doppler spreads from the bulk-Doppler). For the STFT, 512 samples are used for every window. As the wing flapping is very well captured by the radars of both frequency bands, a longer window length is not necessary. The strong zero-Doppler signal is due to the perch and the pole on which it was mounted.

**Figure 6:**
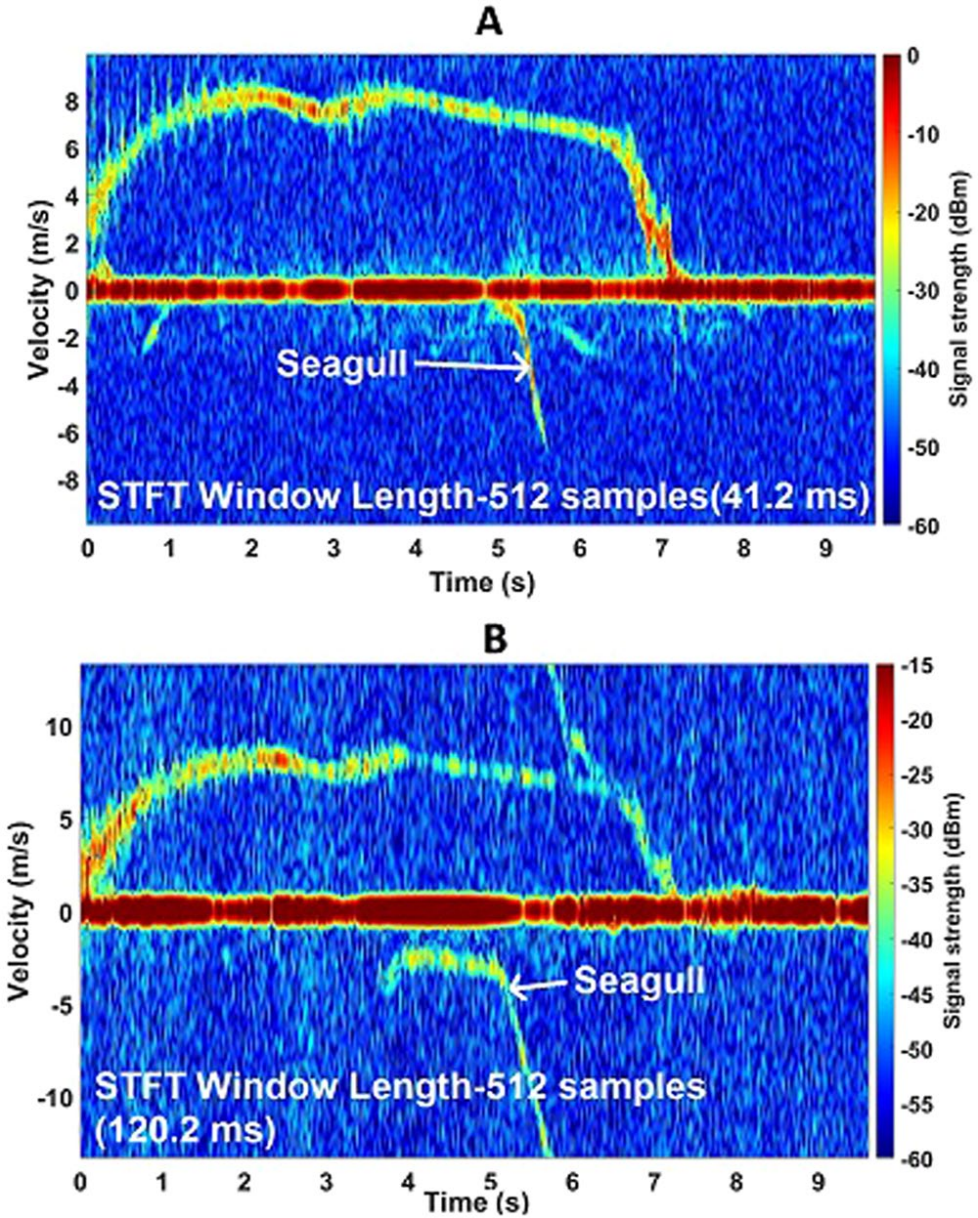
Harris Hawk spectrogram plots showing micro-Doppler features at both K-band and W-band. (**A**) Spectrogram of Harris Hawk flying towards the perch at 85 m away from the radar at 94 GHz, a seagull coming into the view during flight; (**B**) Spectrogram of Harris Hawk towards the perch at 85 m away from the radar at 24 GHz.

**Figure 7:**
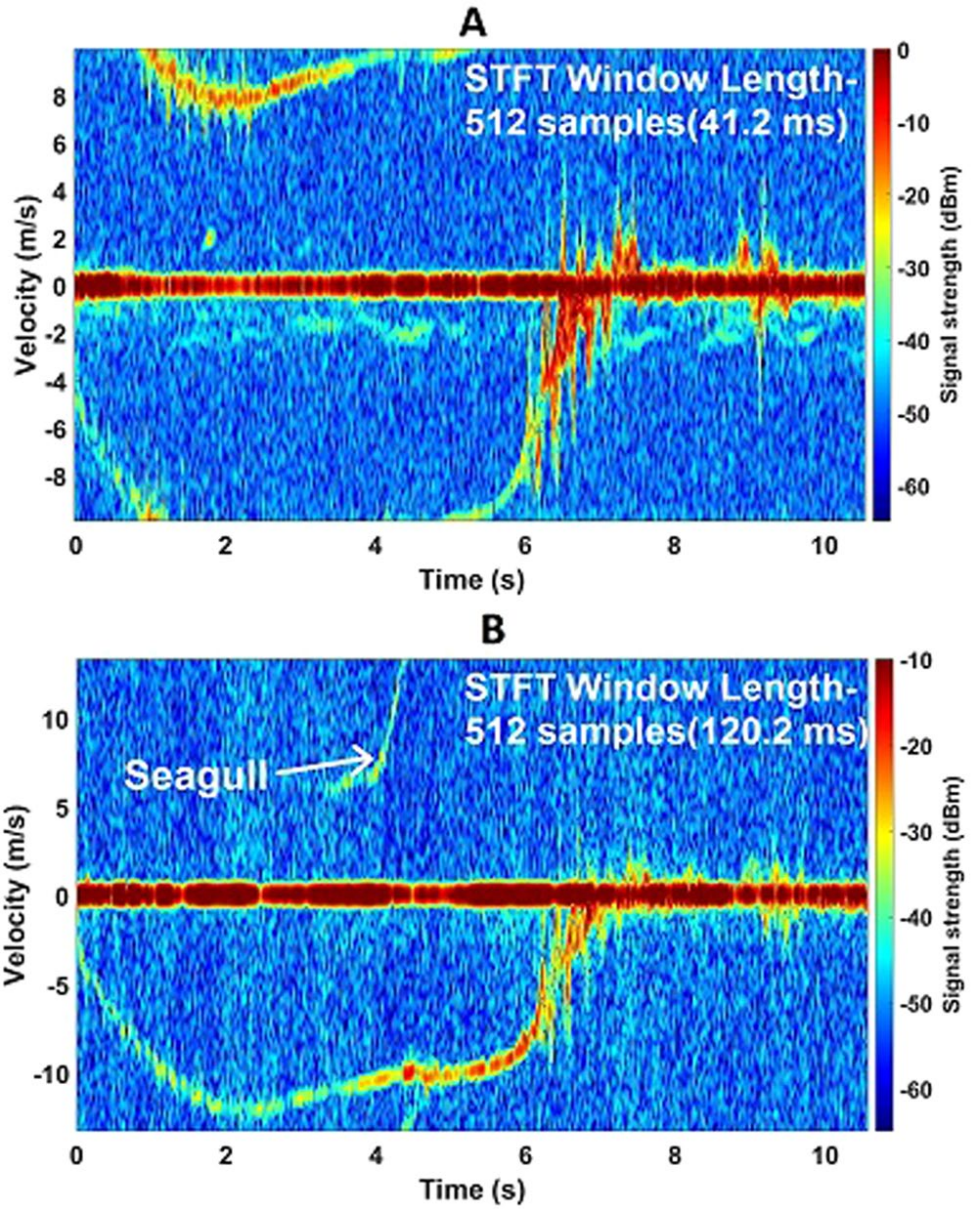
Indian Eagle Owl spectrogram plots showing micro-Doppler features at both K-band and W-band. (**A**) Spectrogram of Indian Eagle Owl flying towards the perch at 30 m away from the radar at 94 GHz, Doppler aliasing is observed; (**B**) Spectrogram of Indian Eagle Owl flying towards the perch at 30 m away from the radar at 24 GHz, seagull coming into the view during flight.

Figure 6(A,B) shows the flight path more in detail for the Harris Hawk which had a straighter trajectory during its flight. The wingbeats at the start of the flight are captured by the 94 GHz radar, seen in Fig. 6(A). No wing beats are seen in the middle when the bird was gliding. During the final approach to the perch, the hawk starts to flap again and the observed wingbeat frequency is around 4 Hz. The wingbeat velocity is about 2–6 ms^−1^. Wingbeats are also seen in the 24 GHz data but not as strongly as the 94 GHz, evident in Fig. 6(B), where the signal strength is generally lower. By coincidence at about 5 seconds, a seagull flew within the radar beam for a very short time then glided away quite quickly without wingbeats and was captured by both radars. Doppler Spectrogram plots of the Indian Eagle Owl at both frequency bands can be seen in Fig. 7(A,B). One perch was 30 m away from the radar and the other one was 100 m away. The data was taken when the bird was flying towards the radar. In Fig. 7(A), it can be noticed that the bird was flying fast enough to be aliased in the measurement. It has negative velocity as it is approaching then the velocity exceeds the maximum limit set for the 94 GHz radar (9.93 ms^−1^), after which the signal appears at the other side of the spectrogram. Wingbeats are also observed with a rate of about 5 Hz. The wingbeat velocity in this case ranges between 2–6 ms^−1^. As observed before with the other birds, this one starts to flap wings again when it approaches the perch. As the velocity range is slightly higher in the 24 GHz radar (13.3 ms^−1^), Doppler aliasing is not observed in Fig. 7(B). The maximum speed the Indian Eagle Owl reaches during the flight is slightly less than 13 ms^−1^. The wingbeats in this case are not observed in the beginning but clearly seen when it reaches the perch. A seagull came into the view of the 24 GHz radar this time which can be seen from the spectrogram.

**Figure 8:**
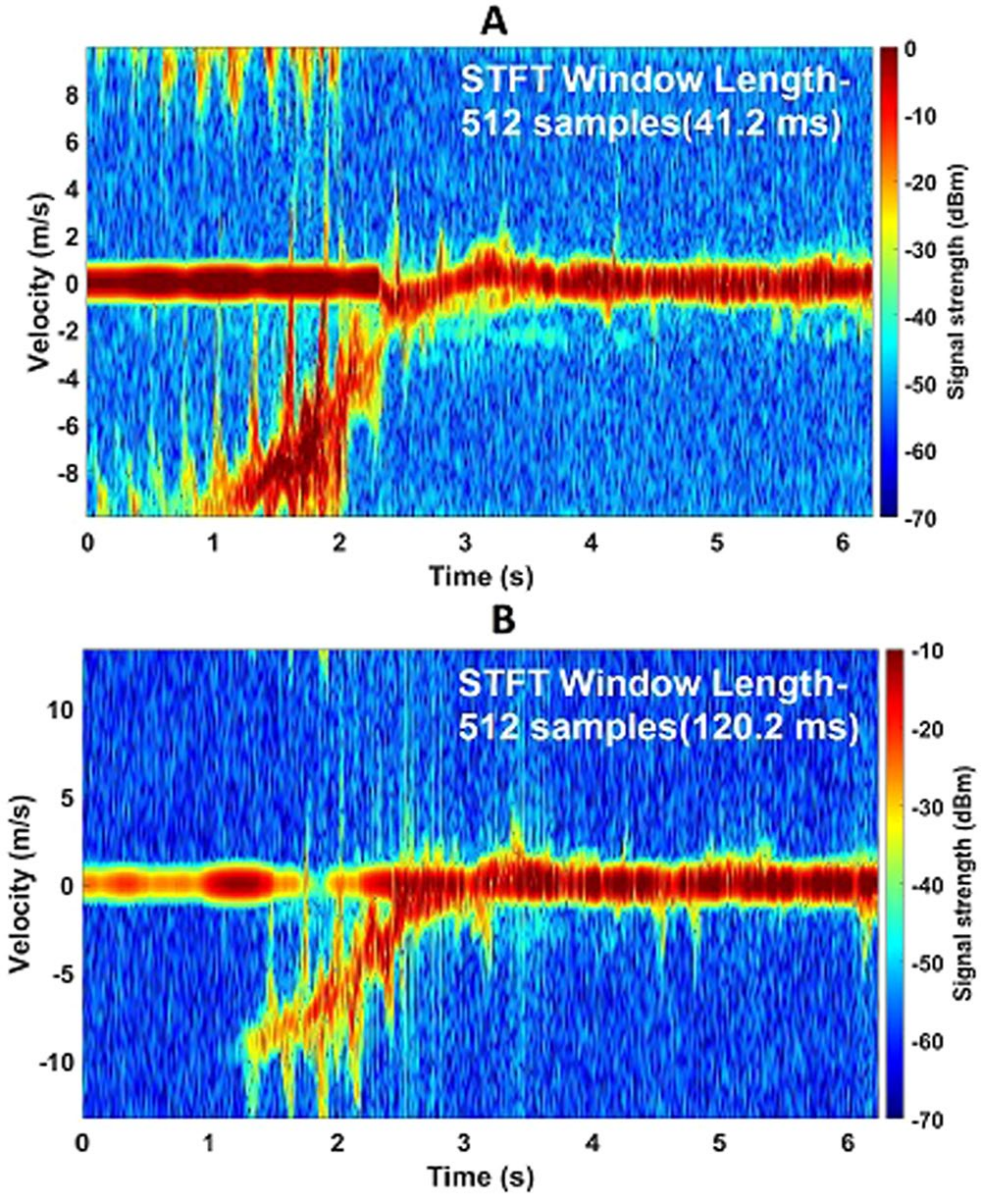
Tawny Eagle spectrogram plots showing micro-Doppler features at both K-band and W-band. (**A**) Spectrogram of Tawny Eagle approaching the perch at 30 m away from the radar at 94 GHz; (**B**) Spectrogram of Tawny Eagle approaching the perch at 30 m away from the radar at 24 GHz. In both cases, strong micro-Doppler due to wingbeats are observed.

Figure 8(A,B) illustrates that the wingbeats of the Tawny Eagle are clearly observed from both 94 GHz and 24 GHz data. The wingbeat frequency is approximately 4 Hz in this case. A large wingbeat velocity is observed in this case, spreading to 11–12 ms^−1^. This bird was the most temperamental among all four hence it was very difficult to have the bird within the radar beam during the entire flight. When it did eventually approach the perch, both radars obtained strong micro-Doppler signals.

It can be seen from all the bird micro-Doppler spectrograms that the difference in the signal strength from the main body and the wings is not as large as the difference between signals from the fuselage and propellers for the drones. This is expected considering the size ratios. The signal return from the wings ranges from being broadly comparable to the main body signal strength (Fig. 8) to approximately 10 dB lower (Fig. 6). An important aspect to consider here is that due to the radial flight path, only the frontal aspect of the bird was seen by the radar. It is expected that the main body/wing signal strength ratio will change if the birds fly across the radar beam. The absolute return power has been observed within the range of 0 dBm to −10 dBm for ranges of approximately 30–85 m, which was well above the radar noise floors.

## Discussion

In this report, micro-Doppler signatures of drones and birds obtained by processing K-band and W-band radar have been illustrated. It is evident from the results that a phase coherent radar system is well suited for capturing micro-Doppler features of these targets, in both frequency bands.

It is very apparent from the results that the micro-Doppler characteristics of drones and birds differ significantly. In the case of drones, the characteristic micro-Doppler signature is the wide signal spread over both sidebands about the bulk-Doppler. Different STFT window lengths have been used along with varying CRF, during drone data collection. This reveals the two main properties of drone micro-Doppler: rotor blade flash and HERM lines, both of which have been observed and shown in this report. It has been established that these micro-Doppler features are characteristic of drones and may be used for classification. All three drones tested have exhibited strong micro-Doppler signatures in both frequency bands with W-band producing a slightly stronger micro-Doppler, about 5–10 dB higher in some cases (Hexacopter, Tawny Eagle), but not all the time. Nonetheless, K-band is also a very good frequency for drone micro-Doppler detection. It should also be kept in mind that the 24 GHz radar has a wider azimuth beamwidth compared to the fan beam antennas of the 94 GHz T-220 radar, so it was easier to keep the drone within the beam during data collection. This is validated by the ability of the 24 GHz radar to generate distinct HERM lines on the spectrogram plots. The DJI Inspire 1 has shown slightly stronger micro-Doppler than the DJI Phantom 3 Standard. Micro-Doppler strength from the Phantom was around −30 dB lower than the bulk Doppler whereas from the Inspire it is around −20 dB lower (there is a large variation in the signal strength due to changing scenario). On the other hand, the DJI S900 Hexacopter micro-Doppler return is very strong throughout the velocity axis. We observed differences of about 10 dB in relative RCS between the small, medium and large drones, at both frequency bands.

In case of birds, there is no such wide micro-Doppler spread and instead in the characteristic signature takes the form of periodic flashes corresponding to the wingbeat frequency. Both the K-band and W-band radar systems have produced high quality spectrogram plots of birds. All species of birds used during data collection produced distinct micro-Doppler signatures. It has been observed that the relative strength of the wing flap micro-Doppler is between 0 and 10 dB below the bulk signal. The measured wingbeat frequencies of all the birds were within the range of 4–6 Hz with the Northern Hawk Owl displaying the fastest wingbeat, consistent with it having the shortest wingspan.

In conclusion, this report provides a catalogue of drones and bird spectrograms, in a way which can be used for any future radar research work considering these targets at K-Band and W-band. It demonstrates that a high-fidelity drone detection radar system is possible in the millimetre-wave band or in K-band. The results can be useful for developing classification algorithms capable of discriminating drones from birds. The rotor blade flash, micro-Doppler spread across the Doppler axis, HERM lines and bird wingbeat signatures could all be used as classification features for reliable target detection. Since the birds are main confusers in a drone detection scenario, the target discrimination algorithm can become more robust by incorporating these different properties shown here during feature extraction. Additionally, the bird data can be used for the design of a bird detection radar at these high frequencies which may find application as a tool for studying bird behaviour.
